# SOCS Proteins Participate in the Regulation of Innate Immune Response Caused by Viruses

**DOI:** 10.3389/fimmu.2020.558341

**Published:** 2020-09-25

**Authors:** Shanzhi Huang, Ke Liu, Anchun Cheng, Mingshu Wang, Min Cui, Juan Huang, Dekang Zhu, Shun Chen, Mafeng Liu, Xinxin Zhao, Yin Wu, Qiao Yang, Shaqiu Zhang, Xumin Ou, Sai Mao, Qun Gao, Yanling Yu, Bin Tian, Yunya Liu, Ling Zhang, Zhongqiong Yin, Bo Jing, Xiaoyue Chen, Renyong Jia

**Affiliations:** ^1^Avian Disease Research Center, College of Veterinary Medicine, Sichuan Agricultural University, Chengdu, China; ^2^Institute of Preventive Veterinary Medicine, Sichuan Agricultural University, Chengdu, China; ^3^Key Laboratory of Animal Disease and Human Health of Sichuan Province, College of Veterinary Medicine, Sichuan Agricultural University, Chengdu, China

**Keywords:** suppressor of cytokine signaling proteins, virus, innate immune, cytokine, TLR

## Abstract

The host immune system has multiple innate immune receptors that can identify, distinguish and react to viral infections. In innate immune response, the host recognizes pathogen-associated molecular patterns (PAMP) in nucleic acids or viral proteins through pathogen recognition receptors (PRRs), especially toll-like receptors (TLRs) and induces immune cells or infected cells to produce type I Interferons (IFN-I) and pro-inflammatory cytokines, thus when the virus invades the host, innate immunity is the earliest immune mechanism. Besides, cytokine-mediated cell communication is necessary for the proper regulation of immune responses. Therefore, the appropriate activation of innate immunity is necessary for the normal life activities of cells. The suppressor of the cytokine signaling proteins (SOCS) family is one of the main regulators of the innate immune response induced by microbial pathogens. They mainly participate in the negative feedback regulation of cytokine signal transduction through Janus kinase signal transducer and transcriptional activator (JAK/STAT) and other signal pathways. Taken together, this paper reviews the SOCS proteins structures and the function of each domain, as well as the latest knowledge of the role of SOCS proteins in innate immune caused by viral infections and the mechanisms by which SOCS proteins assist viruses to escape host innate immunity. Finally, we discuss potential values of these proteins in future targeted therapies.

## Introduction

Host cells have gradually evolved multiple cellular signaling networks to detect and respond to viral infections (1). When the host's PRRs binds to PAMP in viral proteins and nucleic acids, it will trigger an antiviral response (2, 3) and the intracellular signaling cascades will be initiated, which results in the activation of transcription factors, including IFN regulatory factors (IRFs) and nuclear factor-κB (NF-κB) (1). These factors, in turn, will promote and induce gene expression, including IFNs and IFN stimulating genes (ISGs), as well as proinflammatory cytokines and chemokines which are involved in the innate regulation and immune responses (4, 5). In the early infection of the virus, the host's innate immunity is the first defense mechanism initiated by the host. Before the body produces more specific adaptive immune system protection, innate immune is a crucial component of preventing virus invasion and reproduction.

During the viral infection process, cytokines trigger and deal with inflammation. However, excessive production of cytokines can cause a cytokine storm and excessive host innate immune response can also damage the body. All in all, the negative feedback loop of cells plays a vital role in maintaining the close relationship between cytokine secretion and inhibition. Therefore, the SOCS proteins with negative feedback regulation ability prevents the excessive secretion of cytokines from harming the host (6). An intracellular protein family is constituted by SOCS1-7 proteins and cytokine-inducible SRC homology 2 (SH2) domain-containing proteins (CIS; also known as CISH), some of which play an essential role in regulating the response of immune cells to cytokines (7–9). These proteins regulate signaling pathways at the intracellular level, effectively and specifically inhibit cytokine and growth factor signaling (10). Cytokines, including interleukins (ILs), IFNs and hematopoietic growth factors, play a vital role in innate immune response, adaptive immunity, inflammatory response and cell differentiation and proliferation (11). Also, they can activate the JAK/STAT signaling pathway. SOCS proteins mainly regulate the signal transduction of cytokines by inhibiting JAK activity or targeting ubiquitinated signal transduction factors, thereby avoiding body damage caused by excessive secretion of cytokines (12, 13). Studies have shown that the expression of SOCS proteins induced by cytokine stimulation can prevent cytokine signal transduction by inhibiting the JAK/STAT pathway (14–17). This review will explore the central role of SOCS proteins in the virus-induced innate immune response. Emphasis will be placed on the mechanisms involved in SOCS proteins regulating virus-induced innate immune responses.

## SOCS Proteins Composition and Function of Each Domain

SOCS protein was first discovered in the mid-1990's and was found to be an inhibitor of cytokine-induced STAT cell signaling pathway (18–21). Up to now, there are eight members in SOCS family, namely CIS and SOCS1-SOCS7, each of them contains a central SH2 domain, a variable-length and divergent amino (N) terminal domain and a carboxyl (C) terminal 40 amino acids (aa) module (22). In particular, a sequence rich in proline, glutamic acid, serine and threonine is called PEST motif. So far, only SOCS1, 3, 5, 7 and CIS in the SOCS family contain this sequence (23, 24), but it has not been found in SOCS2, 4 and 6 (25, 26). Among the domains that constitute the SOCS protein, the C-terminal 40 aa module is called SOCS box. Studies have shown that it binds to elongin BC in a manner similar to the von Hippel-Lindau protein BC box and shows extended structural conservation with the F box of the Skp2 ubiquitin ligase (27). The SOCS box recruits E2 conjugating enzyme by interacting with elongin BC, cullin-5 and the RING-box-2 (RBX2), which is necessary to complete the negative regulatory process of cytokine signaling (28, 29). SOCS1 can inhibit the carcinogenic activity of TEL-JAK2 and this function requires the participation of SOCS box and kinase inhibitory region (KIR) (30, 31). In addition, mice lacking SOCS1 completely exhibit similar inflammatory diseases as mice with genetically modified the SOCS box of SOCS1 or mice only lacking the SOCS box of SOCS1 (32). However, the specific role and function of the SOCS box still need further exploration. The SH2 domain at the center of the SOCS proteins determines the target of the SOCS family and except for single-cell fungi, the SH2 domain in most eukaryotes is conserved (33, 34). Most importantly, the SH2 domain interacts with the substrate by recognizing phosphorylated tyrosine residues and enhances substrate interaction through the N-terminal extended SH2-subdomain (ESS) (22). The SH2 domain of various SOCS proteins also perform different functions. For example, the SH2 domain of SOCS1 directly binds to the activation loop of JAK, while the SH2 domains of SOCS2, SOCS3 and CIS can only bind to phosphorylated tyrosine residues on the receptor (14). Although the SH2 domain of SOCS3 does not have high affinity for JAK compared to SOCS1, its KIR structure has a higher affinity for JAK2 than SOCS1 (35). SOCS1 can directly bind to IFN-I receptors, thereby ensuring that SOCS1 has a very effective inhibitory effect on IFN signaling even at low expression levels (36, 37). In addition, recent studies have shown that although SOCS1 is an effective inhibitor of the IFN-gamma (IFN-γ) pathway, it cannot directly bind to the IFN-γ receptor (38). Unlike other cytokines that suppress the IFN-I response, SOCS1 is related to IFN-I receptors (IFNAR1) specific signals, but not to IFNAR2 specific signals, thereby eliminating tyrosine phosphorylation of the transcription factor STAT1 and reducing antiviral genes duration of expression (39).

Structurally, we can subdivide the SOCS family according to aa residues. The longest aa sequence in the N-terminal region is SOCS4-7 and the shortest is CIS, SOCS1-3 (12, 40, 41). So far, the most distinctive members of the SOCS family are CIS, SOCS1-3 (42). CIS and SOCS2 compete with STATs, or sterically block the binding site of STATs on the receptor, thereby inhibiting the activation of STATs, such as STAT5 (18, 43). Analysis of SOCS2 knockout (KO) mice uncover that SOCS2 proved to be a relatively specific negative regulator of GH-STAT5 (44, 45). SOCS protein not only has the ability to inhibit signal transduction through ubiquitin-mediated degradation of signal transduction compounds. Studies have shown that SOCS1 and SOCS3 also directly inhibit JAK activity through their KIR domains, which are considered to be pseudo-substrate functions and are essential for inhibiting cytokine signaling (46). The KIR of SOCS3 can block the association of substrate and JAK2 by covering the substrate-binding groove of JAK2. Data show that SOCS1 is an efficient inhibitor of JAK1 and JAK2, TYK2, but does not work on JAK3 (38). This can be account for the SH2-KIR domains interacting with the evolutionarily conservative “GQM” sequence, which is present in JAK1, JAK2 and TYK2 in all vertebrates, but not in JAK3 (47). Besides, a KIR and KIR mimic peptide called tyrosine kinase inhibitor peptide (TKIP) can inhibit the phosphorylation of its downstream transcription factor STAT1 by inhibiting JAK2 signaling (48, 49). In short, revealing how the SOCS protein domains combine or interact with external factors may provide a prerequisite for us to better understand how the SOCS family participates in immune regulation and it also provides a much-needed theoretical basis for biomedical treatment.

## Signaling Pathways Involved in SOCS Proteins

### SOCS Proteins Regulates JAK/STAT Signaling Pathway

**Figure 1:**
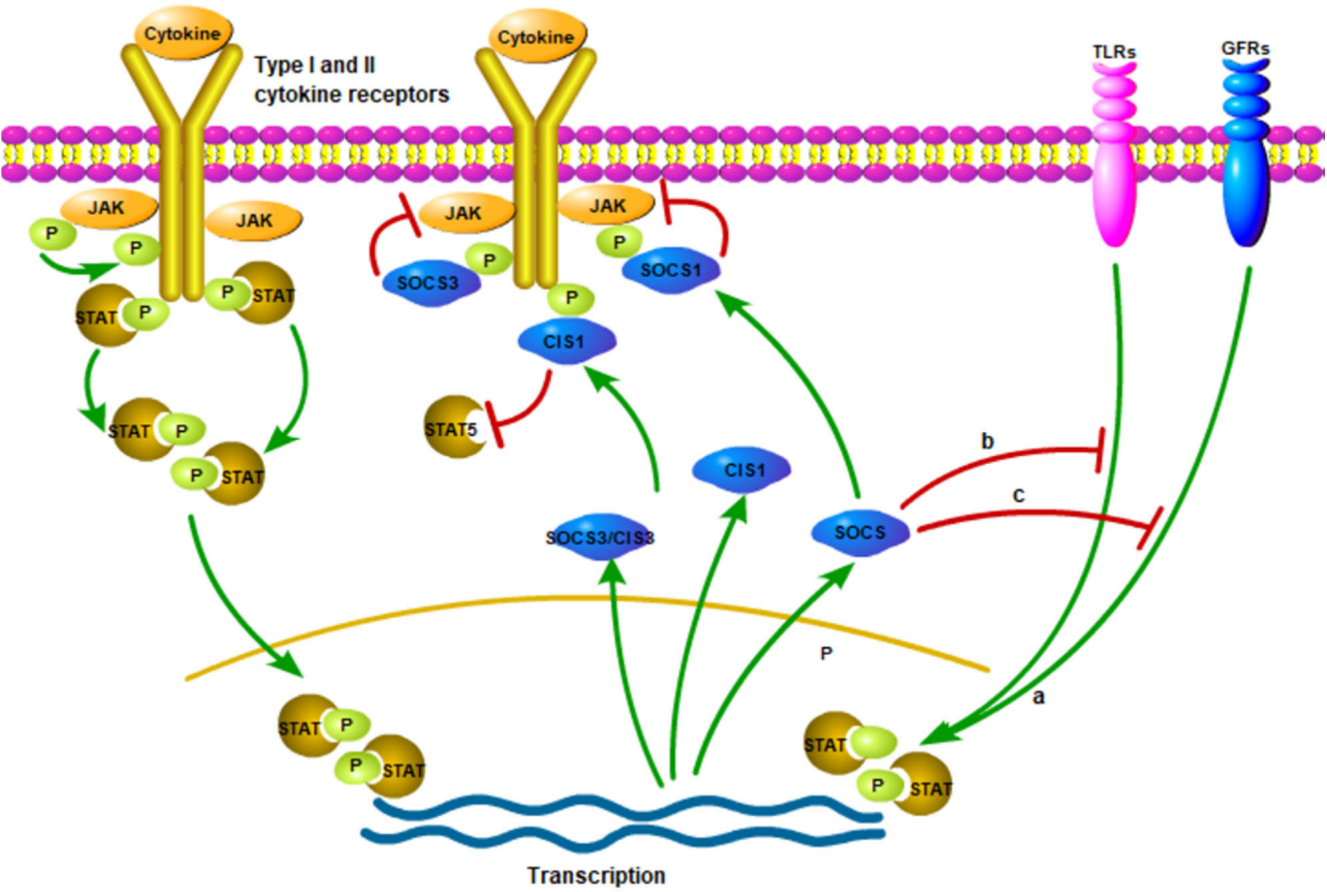
The production and action of SOCS proteins. Various cytokine receptors can induce SOCS proteins expression in a JAK/STAT-dependent manner (Pictured on the left side). However, SOCS1, SOCS3 and CIS seem to suppress signaling in various ways. SOCS1 directly interacts with JAKs and reduce their catalytic activities while SOCS3 interacts with the proximal position and CIS blocks up with the binding sites of STATs to receptors. Moreover, it is possible to induce the production of SOCS proteins independent of JAK/STAT signaling pathways, such as signaling of TLRs, growth factor receptors (GFRs). (a) The SOCS proteins induced in these ways could inhibit the response of the cell to other cytokines, which is one of the modes of crosstalk inhibition. Finally, the inhibition of SOCS proteins may not be limited to the classic JAK/STAT signaling pathway (b,c). The figure is referenced from Figure 1 of Suppressors of cytokine signaling and immunity (14). The red line indicates inhibition; green line indicates promotion; P (in the green circle) indicates phosphorylated.

Once cytokine stimulation occurs, the SOCS proteins will similarly inhibit signal transduction through the JAK/STAT pathway and target the degradation of signal transduction intermediates to prevent further signal transduction in the classical feedback loop (15, 50, 51) (Figure 1). For example, SOCS1 can regulate the activation of M1-macrophages by inhibiting JAK2/STAT1 and TLR/NF-κB signaling pathway induced by IFN-γ (52, 53) (Figure 1). In addition, SOCS3 inhibits kinase activity by binding to JAK2, thereby negatively regulating cytokine signaling (54, 55) (Figure 1). The analysis by different research groups also show that SOCS3 determines the specificity of IL-6 signaling in macrophages (22, 56, 57). Experimental macrophages with deletion of SOCS3 gene will lead to the early induction of STAT1-dependent genes instead of the early induction of STAT3-dependent genes. Therefore, IL-6, in this case, induces STAT1 and STAT3 and partially replicates the activity of IFN-γ. Moreover, SOCS3 ablation leads to the extension and persistence of STAT3 signals. Gene silencing or reducing SOCS3 expression may be the reason for such high STAT3 activity in tumors (58, 59). In addition, CIS can negatively regulate cytokines that signal through the JAK/STAT5 pathway, such as erythropoietin, prolactin and IL-3 receptors (60–62) (Figure 1).

The receptor protein will undergo a conformational change after receiving an external signal, causing a series of physiological and biochemical reactions in cell, which are gradually amplified by the intracellular signal transduction pathway. In mammals, the JAK/STAT pathway is the main signaling mechanism of many cytokines (63). The specific process is as follows: In JAK/STAT signal transduction, JAKs (JAK1, JAK2, JAK3 and TYK2) are activated when various ligands bind to cell surface receptors and form mutually phosphorylated dimers. Phosphorylation activated JAK further phosphorylates cell receptors. Once STAT attaches to the receptors, it is phosphorylated and dimerized by JAKs and then translocated into the nucleus to bind to a specific sequence in DNA (64) (Figure 1). STAT inactivation occurs by dephosphorylating proteins along signaling pathways. External factors that interfere with the signal transduction of the JAK/STAT pathway will affect the body's normal immunity, inflammation and cell proliferation, differentiation and apoptosis (63). In addition, failure to properly activate JAK signaling or mutation signaling-related molecules may lead to inflammation or immune diseases (63).

### SOCS Proteins Regulate TLR Signaling Pathway

PRRs, especially TLRs, identify conserved microbial structures and activate macrophages and dendritic cells (DCs). It has been proposed that SOCS2 may be one of the feedback regulation inhibitors of TLR signal activation in DCs (65). More and more evidence shows that SOCS proteins have a broader function in the regulation of TLR signaling (66, 67) (Figure 1). Moreover, some studies using genetically disrupted KO mice unexpectedly found that SOCS proteins also play an essential role in many immune and pathological processes (42).

### SOCS1 and TLR Signaling

The regulation of TLR signaling is a critical step in the inflammatory response, identifying pathogens and establishing active acquired immunity. Host immune cells have evolved corresponding negative regulatory mechanisms, such as SOCS proteins, to control the excessive immune response caused by collective long-term exposure to LPS. In macrophages, stimulation of CpG-DNA or Lipopolysaccharide (LPS) induces the production of SOCS1 and SOCS3 (50, 68, 69). SOCS1 protects the host from the lethal LPS response and this has been verified in a model of SOCS1-deficient mice (66, 70, 71). Studies have shown that SOCS-deficient mice are highly sensitive to LPS stimulation through myeloid differentiation factor 88 (MyD88) -dependent and independent pathways related to IL-1-receptor-associated kinase (IRAK1) (17, 72–74). In addition, SOCS proteins also play a key role in the innate immunity caused by viruses involved in TLR signals. The viruses escape innate immunity by using the negative regulation of SOCS proteins on cytokines. On the one hand, SOCS proteins can regulate TLR-mediated signal transduction. On the other hand, TLR signaling can, in turn, regulate SOCS proteins expression in various kinds of cells (22, 75). For example, in pDC cells, hepatitis B virus (HBV) can use SOCS1 up-regulation to inhibit TLR9-mediated IFN-α production, thereby inhibiting intracellular antiviral responses and promoting virus replication (76). Studies have shown that the activation of TLR7 in human plasmacytoid DCs can induce the expression of SOCS1 and SOCS3, while SOCS1 and SOCS3 will strongly inhibit TLR7-mediated IFN-I production (77). In addition, Yu Peng and his colleagues demonstrated that IRF7 combined with SOCS1 and SOCS3 and the SH2 domains of SOCS1 and SOCS3 promoted proteasome-mediated degradation of IRF7 through polyubiquitination related to lysine 48 (77). Moreover, studies have shown that TLR8 couples with SOCS-1 to control the TLR7-mediated antiviral immunity in the mouse central nervous system during West Nile virus (WNV) infection (78). It has also been studied that the binding of hepatitis C virus (HCV) core protein and complement receptor gC1qR on monocytes/macrophages (M/MFs) triggers the expression of PD-1 and SOCS-1, which can provide negative signals to the TLR-mediated IL-12 expression regulation pathway and IL-12 is a key cytokine connecting innate and acquired immunity (79). The study also showed that the SOCS1 transcript of goose was induced by goIFN and TLR ligands in GEF cells and PBMC, respectively. It is worth noting that a high expression level of goose SOCS1 (goSOCS1) was detected after infection with high viral load of Duck Tembusu virus (DTMUV) *in vitro* and *in vivo*, which suggests that goSOCS1 may be related to innate and adaptive antiviral immunity (80). The cytokine response is well-regulated by a variety of homeostatic mechanisms, including microRNAs (miRNAs) that can quickly target specific genes involved in the control of immune signaling pathways. Studies have shown that there are several immune-related miRNAs differentially expressed in monocyte-derived macrophages (MDM) and vitamin D3-treated MDM (D3-MDM) after dengue virus infection. It is worth noting that miR-155-5p plays a major role in the cytokine response induced by TLR. The attenuation of miR-155-5p in D3-MDM was confirmed to be related to the increased expression of its target gene SOCS-1. In addition, D3-MDM differentiation induced the down-regulation of surface TLR4, which was related to the decreased secretion of IL-1β derived from TLR4/NF-κB (81).

**Figure 2:**
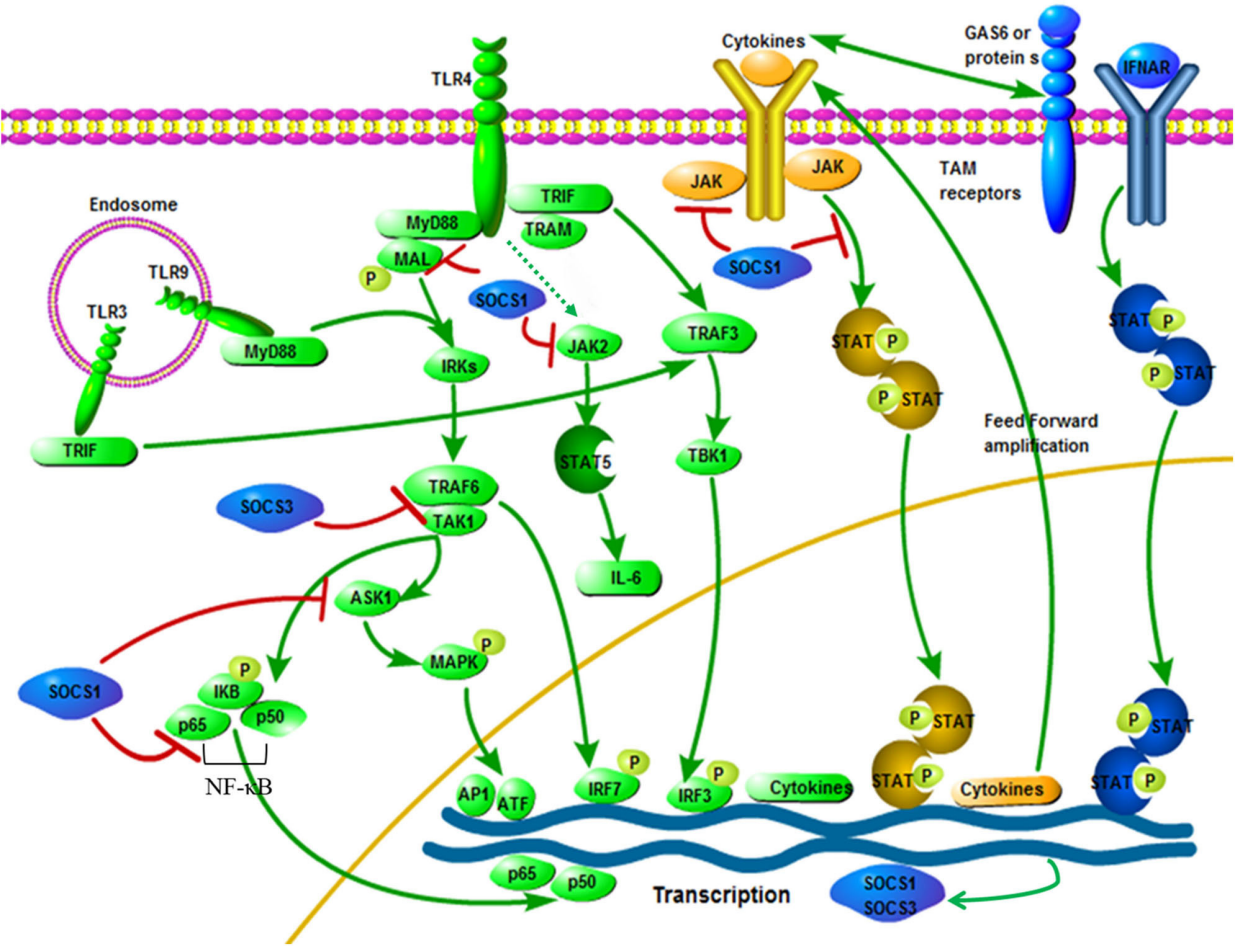
Interactive roles of SOCS1 and SOCS3 in TLR signaling. Pathogen exposure stimulates static cells, which activate the TLR signaling pathway (green). This leads to an initial outbreak of the proinflammatory cytokine, which are then greatly amplified in the feedforward loop through the cytokine receptor signaling pathway (yellow). Cytokine signaling also induces the up-regulation of TAM receptor expression, thereby driving TAM receptor signaling. Besides, TAM receptor signaling requires a synergistic interaction between IFNAR and transcription factor STAT1 (blue). The expression of SOCS1 and SOCS3 are induced, which widely inhibits the cascade of TLR and cytokine receptors, thereby ending the innate immune response (blue). SOCS1 and SOCS3 specific ways to inhibit TLR signaling: After LPS activates TLR4, signals are transmitted through the adaptor proteins including MyD88, MAL, TRIF and TRAM. Through MyD88 and MAL, NF-κB and MAPKs are activated by TRAF6 and TAK1, while IRF3 is activated by TRAM and TRIF. The TRIF-IRF3 pathway can rapidly induce the production of cytokine and activate the JAK/STAT signaling pathway. The JAK2/STAT5 signaling pathway is activated by LPS, which is connected with the IL-6 production. Besides, SOCS1 can inhibit the signaling of these JAK/STAT pathways. Phosphorylated MAL is connected with SOCS1, resulting in polyubiquitination and degradation of the MAL. Moreover, interactions between SOCS1 and p65 subunit of NF-κB can induce its degradation process to inhibit signaling. Besides, NF-κB-dependent transcription is inhibited by SOCS3 by binding inhibition between TRAF6 and TAK1. The figure is referenced from Figure 2 of Immunobiology of the TAM receptors (85). IRAK, IL-1-receptor-associated kinase; TAK1, transforming growth factor-β activated kinase 1; AP1, activator protein 1; GAS6, growth arrest-specific 6. Red line indicates inhibition; green line indicates promotion.

Several mechanisms for inhibiting cytokine production through SOCS1 have been proposed. The first is that SOCS1 directly affects the signal transduction of the TLR/NF-κB signaling pathway (66, 70). SOCS1 attaches to the p65 subunit of NF-κB and promotes its degradation mediated by ubiquitination. SOCS1 also interacts with Bruton tyrosine-kinase (BTK) to bind to the tyrosine phosphorylated MyD88 adapter-like protein, also known as TIRAP and induces MAL ubiquitination and degradation, which ultimately leads to inhibition of MAL dependence p65 phosphorylation and NF-κB transactivation (39). SOCS1 not only regulates the NF-κB signaling pathway, but also regulates the gene expression of MAPK, JUN N-terminal kinase (JNK) and p38 that are activated by stress by binding to apoptosis signal-regulated kinase 1 (ASK1) (82). Furthermore, the critical mechanism by which SOCS1 inhibits activated macrophages is by inhibiting the secondary activated JAK/STAT pathway (72, 83). TRIF-IRF3 pathway can quickly induce IFN-β expression and activate JAK/STAT1 within 1 h after stimulation and then promote the expression of CD40 and other genes with NF-κB. SOCS1 is one of the key inhibitors of this signaling pathway (84) (Figure 2). In addition, it is reported that LPS/TLR4 can activate the JAK2/STAT5 pathway and that the activation of this signaling cascade leads to the massive secretion of pro-inflammatory cytokines TNF-α, IL-6, IFN-β and RANTES, while SOCS1 can be selectively inhibit the JAK2/STAT5 pathway to inhibit the secretion of IL-6 (67, 86, 87) (Figure 2). It has been reported that SOCS1 regulates IFN-β-induced JAK/STAT pathway by directly inhibiting STAT1 phosphorylation and indirectly inhibiting TLR4 signaling through IRF3 (88).

### SOCS3 and TLR Signaling

SOCS3 is one of the inducible proteins with abundant expression in macrophages after LPS stimulation and it has been proved that it is also a pivotal regulator of the different levels of activity of IL-6 and IL-10 after TLR stimulation (89, 90). In addition, LPS-induced STAT1, STAT3 and IL-6 expression increased in SOCS3-deficient macrophages, but had no effect on the activation of NF-κB and ERK1/2 (88, 91). It is generally believed that SOCS3 can indirectly affect the TLR signaling pathway through STAT3 activation, but SOCS3 itself may be one of the mediators of the anti-inflammatory response, because both IL-10 and prostaglandin E2 (another potent anti-inflammatory mediator) can induce the up-regulation of SOCS3 protein levels through the cAMP pathway (92). SOCS3 may have a certain effect on the TLR signaling pathway, but this statement is still controversial (93). However, recent studies have shown that SOCS3 can act on the key molecules of TLR signaling and participate in the regulation of TLR signaling. First, at the physiological level, ectopic expression of SOCS3 can inhibit the LPS-mediated expression of TNF and CD40 genes in macrophages (94, 95). Secondly, studies have shown that SOCS3 promotes TLR4 response through feedback inhibition of endogenous transforming growth factor-beta1 (TGF-β1)/Smad3 signaling (96). Moreover, there are also reports that SOCS3 can inhibit the activation of tumor necrosis factor receptor associated factor 6 (TRAF6) and TGF-β activated kinase (TAK1), which are key factors for the induction and signaling of TLR and IL-1 (52) (Figure 2). Recent studies on acute pancreatitis have also shown that LPS up-regulates the expression levels of SOCS1 and SOCS3 in acinar cells, while SOCS1 and SOCS3 directly interact with TRAF6 and degrade TRAF6 protein through polyubiquitination, thereby counteracting the protective function of TRAF6 in the inflammatory response (97). Mechanism characterization indicated that Ezh2 deficiency directly stimulates SOCS3 expression, thus enhancing Lys48-linked ubiquitination and TRAF6 degradation. As a result, in the absence of Ezh2, TLR-induced MyD88-dependent activation of NF-κB and the expression of pro-inflammatory genes in macrophages/microglia are impaired (98). In addition, studies have shown that MRL-1pr/lpr mice (a Th1-mediated autoimmune disease model) after oral administration of HA900 (a high molecular weight hyaluronic acid) can up-regulate SOCS3 expression in colorectal epithelial cells through TLR4, thereby regulating Th1-mediated autoimmune diseases and inflammation (99). Therefore, SOCS3 may indeed have a regulatory role in TLR signaling. In recent studies, it has been discovered that the cyclic GMP-AMP (cGAMP) synthase (cGAS)-IFN gene stimulator (STING)-mediated cytoplasmic DNA sensing pathway that exists in parallel with TLR9-mediated DNA recognition can also induce the production of SOCS1 and SOCS3, thereby activating the IFN-I inhibitory loop, which indicates that SOCS3 may participate in innate immunity through other ways (100).

### SOCS Proteins Regulate Other Signaling Pathways

SOCS protein is not limited to regulating the classic JAK/STAT signaling pathway, but can also inhibit the signal transduction of cytokines such as insulin and Toll-like receptors and none of them activate the JAK/STAT pathway (101). SOCS4 and 5 regulate epidermal growth factor receptor (EGFR) signaling, of which SOCS5 can regulate viral replication by down-regulating EGFR signaling (40). The data suggests that SOCS3 may act directly by preventing JAK activation or mediating ubiquitination of cytokine/growth factor/hormone receptors and subsequent proteasome degradation. Moreover, SOCS3 has been shown to bind to indoleamine dioxygenase (IDO) and mediate the ubiquitination of the complex in DCs. Therefore, SOCS3 antagonizes the IDO-dependent tolerogenic signal in DCs at the post-transcriptional level and converts it into immunogenicity (102, 103). SOCS3 can also bind and degrade CD33 (a myeloid cell differentiation antigen) or Siglec3, thereby blocking the proliferation inhibition caused by CD33 (104). In addition, SOCS3 can regulate the body's sensitivity to insulin by binding and targeting proteasome degradation of insulin receptor (IR) or insulin receptor substrate 1 (IRS-1) (105–108). It is reported that SOCS3 can also directly interact with SMAD3, thereby inhibiting the response to transforming growth factor-β (TGF-β) (96). On the other hand, there are reports that TGF-β can induce the expression of SOCS3, thereby promoting the formation of osteoclasts induced by TNF (109). Unexpectedly, it was also found that phosphorylated SOCS3 binds to IκB (NF-κB inhibitor), hindering its degradation and thus preventing the activation of NF-κB (110). Studies have also shown that SOCS3 can block IL-1 induced NF-κB and JNK/p38 signaling pathways by binding and inhibiting the signaling of upstream molecule TRAF6 (52). Taken together, the SOCS family are key factors in the feedback regulation of cytokine signaling pathways. Therefore, they play a vital role in regulating inflammation, immunity, growth regulation and determining cell fate.

## SOCS Proteins Regulate Innate Immunity Caused by Viruses

### Virus-Induced Innate Immune Process

During innate immunity, PRRs are involved in identifying and detecting specific viral components and activates corresponding immune signaling pathways in infected cells and other immune cells to enhance the secretion of inflammatory cytokines and IFN-I (111). IFN-α/β is the principle cytokine that limits virus replication, while other cytokines, including TNF-α, IL-1β and IL-6, recruiting immune cells to the site of infection and causing an inflammatory response. Therefore, it is important to distinguish between self and non-self nucleic acids, especially during viral infection. Recent advances in innate immunity research indicate that this distinction is mostly dependent on PRRs, including the TLR, RIG-I like receptor (RLR) and nucleosides acid-binding oligomeric domain (NOD) like receptor (NLR). These innate immune receptors trigger signaling cascades that are usually integrated with innate responses, such as NF-κB dependent cytokine responses, IRF dependent IFN-α/β responses, inflammatory body/caspase-1 dependent IL-1β responses. NF-κB-dependent and IRF-dependent cytokines are regulated by transcription, while inflammatory body-dependent IL-1β secretion is regulated by transcription and post-transcription (112).

Among various PRRs, TLRs have now been studied in depth. Today, a complex picture of TLR signaling has emerged (113, 114). The critical fact is that microbial stimulation directly binds to TLRs, causing their conformations to change. Among them, heterodimers (TLR1, 2, 6) or homotypes (TLR3, 4, 9) are activated and related to intracellular adaptor molecules. Except for TLR3, the central adaptor of all other TLRs is MyD88. For TLR2 and 4, the adaptor/MyD88-adapter-like protein (TIRAP/Mal) containing the TIR domain promotes this binding. In particular, TLR3 and TLR4 can further bind to the Toll/IL-1 receptor domain-containing adaptor inducing IFN-β (TRIF), so they can also use TRIF-related molecules (TRAM) as adaptors. Finally, MAPK and NF-κB signaling related molecules are activated through MyD88-dependent signaling (Figure 2). TRIF mainly activates the transcription of IRF3 gene through another pathway, thereby inducing IFN-β production and then sends signals in an autocrine or paracrine manner (115). It has also been proposed that other signaling modules are involved in TLR signaling and they are particularly important to activate IRFs family members with specific cell types and ligands way (116). However, once these receptors are activated, corresponding negative feedback regulation will occur to avoid innate immune overreaction. Under such circumstances, the SOCS proteins will exert their negative feedback regulation effect.

### Virus Hijacking SOCS Proteins Escapes Innate Immunity

Cytokines activate the innate immune response and initiate a specific immune response against viruses, but incorrect activation of the immune response can lead to dysregulation of host cytokine signaling during disease infection and ultimately cause tissue and organ dysfunction. The SOCS family are a class of negative regulators induced by cytokines, which can block the signal transduction of cytokines, thereby avoiding the body's excessive immune response (117). SOCS1 overexpression leads to a decrease in the phosphorylation levels of JAK1, TYK2 and STAT1 and inhibits the antiviral and antiproliferative responses induced by IFN-I (118, 119). Many innate or adaptive immune transcription factors can induce the production of SOCS proteins, but under normal physiological conditions, the most significant SOCS proteins production is induced by cytokines such as IFN-I, IFN-II and IL-6 through the JAK/STAT signaling pathway (21, 120–123). SOCS1 ablated cells and SOCS1^−/−^ mice are resistant to viral infection (37). It seems that the negative regulation of other cytokine pathways is the main role of SOCS3, such as the regulation of IL-6 family cytokine signaling (55), but it has also been shown that SOCS3 can inhibit the antiviral function of IFNs.

Due to the immunomodulatory effect of SOCS proteins, it is normal to think that infectious micro-organisms can manipulate the host's SOCS proteins to escape innate immunity. Studies show that Porcine Reproductive and Respiratory Syndrome virus (PRRSV) can choose SOCS1 to evade the host's immune response and SOCS1 can inhibit the expression levels of ISGs and IFN-β to promote viral replication (124). Furthermore, when HCV and herpes simplex virus 1 (HSV-1) infect human hepatoma cells and amniotic cells, respectively, they can abrogate IFN-α/β signaling by enhancing SOCS3 protein expression (125, 126). Influenza virus infection mainly inhibits JAK/STAT pathway signaling by up-regulating the expression of SOCS1 and SOCS3, thereby disrupting the host antiviral defense mechanism mediated by IFN-I and IFN-II (127, 128). Studies have shown that the non-structural protein 1 (NS1) of respiratory syncytial virus (RSV) inhibits the antiviral response induced by IFN and the production of chemokines by inducing the up-regulation of SOCS1 and SOCS3 (129). The SOCS proteins have been extensively studied and have been known for many years to induce SOCS proteins by activating cytokine receptors, primarily through the activation of IFNAR (42, 130). For example, the production of a large amount of inflammatory factors during influenza A virus (IAV) infection leads to the expression of SOCS1 and SOCS3 through RIG-I/MAVS/IFNAR1-dependent pathways, which ultimately inhibited the antiviral response (131). Moreover, TLR-mediated IFNAR-STAT1 signaling leads to the up-regulation of TAM (Tyro3, Axl and Mer) receptor tyrosine kinases, which in turn tampers with the IFNAR-STAT1 box and ultimately induces the expression of SOCS1 and SOCS3 to suppress cytokine and TLR signals transduction (132). This mechanism also exists in the innate immunity caused by the virus. During Zika virus (ZIKV) infection, Axl regulates the expression of SOCS1 in a STAT1/STAT2-dependent manner, thereby antagonizing IFN-I-mediated antiviral immunity, promoting virus infection and replication (133). Similarly, enveloped viruses such as WNV can disrupt the DC's innate immune response through the negative regulatory mechanism of IFN-I signaling mediated by AXL-SOCS1 (134). Thus, viruses can inhibit IFNAR signaling through the inhibitory protein SOCS1 through AXL and other TAM receptors, hence evading the innate immune response pathway activated by the TLR receptor and promoting its infection (85). The SOCS proteins not only regulate the production of IFN-I and IFN-II but also IFN-III. Although the host secretes IFN-I and IFN-λ during viral infection to suppress viral infection. Nevertheless, the main IFN induced in the nasal epithelium during respiratory virus infection is IFN-λ instead of IFN-I and its production can also help prevent respiratory virus infection (135). Studies have shown that the inhibition of IFN-λ signal by SOCS-1 induced by influenza virus leads to an increase in the adaptability of host IFN-λ expression, thereby protecting cells from virus infection, but it leads to the overproduction of IFN-λ, which ultimately results in the sabotage of antiviral response (136). Recent studies have also shown that IL-17A attenuates IAV-induced IFN-λ expression by enhancing the expression of SOCS1 and SOCS3 to inhibit the autocrine signaling circuit in human airway epithelial cells (137). Similar reports have been reported in flaviviruses, ZIKV infection causes up-regulated expression of SOCS1 and SOCS3, thereby inhibiting RLR dependent IFN-I and IFN-III secretion, indicating that in ZIKV infection, SOCS proteins may regulate viral replication by modulating the antiviral innate immune response (138). In fact, immune evasion of various viruses, such as HBV (139), HCV (79, 125), human immunodeficiency virus (HIV) (140–143), Semliki forest virus (37), coxsackievirus (144), RSV (145), Ebola virus (146), IAV (147), HSV-1 (126, 148, 149), Varicella-zoster virus (VZV) (150), Japanese encephalitis virus (JEV) (151) and Epstein-Barr virus (EBV) (152) by targeting SOCS1 and/or SOCS3 has been reviewed elsewhere (153, 154). Studies have also shown that in HCV infection, HCV protein P7 mediates the up-regulation of SOCS3 through the JAK/STAT signaling pathway and MAPK pathway, while up-regulation of SOCS3 suppresses TNF-α-mediated IκB degradation and subsequent NF-κB promoter activity, which leads to the inhibition of the inflammatory response during HCV infection (155, 156). HSV is the second virus found to induce the production of host SOCS3 protein (126). Studies have shown that JAK signal will stimulate the expression of SOCS3 in HSV-infected FL cells and the overexpression of SOCS3 will down-regulate the antiviral effect mediated by IFN-α/β signals, thereby promoting virus replication (148) (Table 1).

**Table 1:** Virus Hijacking SOCS Proteins Escapes Innate Immunity.

**Virus**	**Target SOCS**	**Effect**
PRRSV	↑SOCS1	↓IFN-β, ISGs ↑viral replication
HCV	↑SOCS3	↓IFN-α/β ↑viral replication
HSV	↑SOCS3	↓IFN-α/β ↑viral replication
IAV RSV WNV ZIKA HCV	↑SOCS1, 3 ↑SOCS1, 3 ↑SOCS1 ↑SOCS1, SOCS3 ↑SOCS3	↓type I and type II IFN, ↑IFN-λ ↑viral replication ↓IFN signal, chemokines, ISGs ↑viral replication ↓type I I IFN ↑viral replication ↓type I and type III interferon ↑viral replication ↓NF-κB, inflammatory response

*↑ increases; ↓ decreases*.

Some recent studies have also explored the effects of SOCS2 gene knockout on viral infection during viral infections such as HSV-1 (157), HSV-2 (158), or bovine herpesvirus type 5 (BHV-5) (159). For example, under the same infection with HSV-1, SOCS2 gene-deficient mice showed less severe immune cell infiltration, encephalitis and neuroinflammation than wild-type C57BL/6 mice (157). Unlike HSV-1 infection, SOCS2 KO mice have worsened meningoencephalitis compared to wild-type animals during BHV-5 infection, indicating that SOCS2 protein has a protective effect on intracranial BHV-5 infection (159). There are also reports on SOCS5 in the regulation of influenza virus infection. After experimental infection with IAV, SOCS5 deficient mice showed more severe viral infections than the control group, such as increased virus titers and weight loss in mice, further research found that SOCS5 may play a vital role in restricting IAV infection of airway epithelium by regulating the EGFR signaling pathway (160). In the future, there may be more viruses affecting SOCS proteins. In general, due to the negative regulation of SOCS in the cytokine signaling pathway, the virus manipulates the expression of various SOCS genes to suppress antiviral signals and evade innate immune responses.

### MicroRNAs Regulate Innate Immunity Through SOCS Proteins

Recently, miRNAs are increasingly considered as an essential factor that regulates the interaction between virus and host (161), many studies have also shown that there is a change in the miRNAs expression profile of the host or virus in viral infections and some of these miRNAs can regulate the expression of SOCS proteins to regulate innate immune pathways. miRNA mainly regulates the expression of immune-related genes in the body by targeting the 3'-untranslated region (3'-UTR) of the target gene, thereby inhibiting or promoting the antiviral response mediated by downstream cascade signals (162–169). On the one hand, the virus itself can generate viral miRNAs, or the virus infection can lead to changes in the expression profile of host miRNAs (170–174). On the other hand, some viral miRNAs and altered host miRNAs can directly target the viral genome or indirectly target host genes to regulate viral replication (175–177). For example, in infectious bursal disease virus (IBDV) infection, the host miR-155 can inhibit the expression of SOCS1 and TANK to promote IFN-I-mediated antiviral response, thereby inhibiting IBDV replication (169). Besides, the authors found that miR-130b of the host can also inhibit IBDV replication by targeting the viral genome and the host's SOCS5 protein, similarly, miR-454 can inhibit IBDV replication by targeting the IBDV genome and SOCS6 (169, 178). In IBDV infection, gga-miR-27b-3p ectopically expressed in DF-1 cells can directly inhibit SOCS3 and SOCS6 and promote the expression of chicken IRF3, NF-κB and IFN-β genes, thereby inhibiting IBDV replication (179). HCV infected cells cause up-regulation of miR-221 expression, which inhibit the expression of SOCS1 and SOCS3, thereby promoting the JAK/STAT signaling pathway and enhancing the anti-HCV effect of IFN (180). JEV infection in human brain microglial cells (CHME3) down-regulated the expression of miR-432, which reduced the phosphorylation of STAT1 and ISRE activity by negatively regulating the expression of SOCS5 and ultimately promoted cell inflammation and virus replication (181). Transmissible gastroenteritis virus (TGEV) infection can cause ER (endoplasmic reticulum) stress and increase inositol-requiring enzyme 1α (IRE1α) expression which downregulates the abundance of host miR-30a-5p that normally enhances antiviral responses by targeting SOCS1 and SOCS3 expression, thus facilitating TGEV replication (182). Moreover, in LTEP-α-2 and SPC-α-1 human lung cancer cell lines experimentally infected with HSV-2, the virally-encoded microRNA Hsv2-miRH9-5p targets and inhibits SOCS2, thereby driving experimental tumor metastasis in these cell lines (158) (Table 2). Taken together, miRNA participates in the immune pathway caused by the virus by regulating the expression of the SOCS gene during viral infection and this also provides new ideas for our future treatment plans and clinical applications.

**Table 2:** MicroRNAs regulate innate immunity through SOCS proteins.

**Virus**	**miRNA**	**Target SOCS**	**Effect**
IBDV	↑ miR-155	↓ SOCS1	↑ type I IFN ↓ viral replication
IBDV	↑ miR-130b	↓ SOCS5	↑ IFN-β ↓ viral replication
IBDV	↑ miR-454	↓ SOCS6	↓ viral replication
IBDV HCV	↑ miR-27b-3p ↑ miR-221	↓ SOCS3, 6 ↓ SOCS1, SOCS3	↑ IFN-β, IRF3 and NF-κB ↑ anti-HCV IFN, ↑ JAK/STAT signaling
JEV	↓ miR-432	↑ SOCS5	↑ phosphorylation of STAT1, ↑ cellular inflammation, ↑ viral replication
TEGV HSV-2	↓ miR-30a-5p ↑Hsv2-miR-H9-5p	↑ SOCS1, SOCS3 ↓ SOCS2	↓ type I IFN ↑ viral replication

*↑ increases; ↓ decreases*.

## Therapeutic Implications

Since SOCS proteins play a key regulatory role in cytokines (especially IFNs), increasing studies have shown that viruses utilize the function of host SOCS proteins to escape the host's immune process, so these proteins also have great therapeutic potential (153). One therapeutic strategy to consider would be suppression of SOCS proteins levels or function during viral infection. A small synthetic peptide containing a phosphorylation activation loop of JAK2 and capable of isolating SOCS is pJAK2, which is also an antagonist of SOCS1 and SOCS3 (48). The peptide has been shown to have therapeutic effects in keratinocytes infected with HSV-1 and can also prevent mice from lethal doses of vaccinia virus, IAV and encephalomyocarditis virus infections (149, 183, 184). Moreover, the use of siRNA to silence SOCS1 in DCs allows these cells to better elicit anti-HIV-1 antibody and T cell responses in mice (140). In addition, miRNAs are increasingly considered to be regulators of SOCS expression (185). For example, studies have shown that inhibiting the expression of miRNA-122 to enhance promoter methylation to inhibit the expression of SOCS3 is a promising option for the treatment of HCV (186–188). Until recently, IL-7 therapy has also been shown to improve the immune response to persistent infections caused by HIV, HBV and HCV by targeting SOCS3 (189). Although these examples are limited and preliminary, they suggest that manipulation of the SOCS proteins may provide an effective mechanism to inhibit viral replication during viral infection. On the other hand, overexpression of SOCS protein through viral vectors may be an effective strategy to inhibit inflammation response. In a study using mice with experimental arthritis, it was found that compared with the control group, injection of recombinant adenovirus carrying SOCS3 cDNA in the joint cavity can significantly reduce the severity of arthritis and joint swelling (190). In addition, in order to suppress the host's inflammatory response, a method of exogenously expressing SOCS1 with therapeutic viral vectors has been proposed (191, 192). This strategy may expand the scope and efficacy of viral vectors in the future.

Since the deletion or inhibition of SOCS1 and SOCS3 in T cells or myeloid cells enhances anti-tumor immunity, SOCS inhibitors may be ideal drugs for targeting immune checkpoints controlled by cytokines (193). One method is to silence the expression of SOCS proteins in DCs or CTL through specific siRNA to enhance anti-tumor immunity (194). But under normal circumstances, silencing the expression of SOCS1 and SOCS3 genes will exacerbate canceration. In addition, SOCS1 gene silencing may promote JAK/STAT signal transduction, which leads to an increase in the body's response to cytokines and ultimately promotes the survival and expansion of myeloma bone marrow cells (195). At the same time, the overexpression of SOCS proteins in tumor cells is another way to inhibit tumor growth by inhibiting tumor-promoting STATs (196, 197). For example, as a mimetic of SOCS proteins, TKIP can effectively inhibit JAK2-mediated STAT1 phosphorylation and proliferation of prostate cancer cells (48, 198). Platelet factor 4 enhances the expression of SOCS3 protein, thereby inhibiting STAT3 activation and inducing apoptosis in myeloma (199). TSA increases the expression of SOCS1 and SOCS3 in human colorectal cancer cells and inhibits the growth of CRC (200). New preclinical data show the potential of using gene therapy to induce SOCS3 overexpression in castration-resistant prostate cancer (CRPC), which may work by attenuating the IL-6-JAK/STAT signaling pathway (201). Taken together, these studies encourage the clinical application of new therapies that modulate SOCS proteins expression or function.

## Concluding Remarks

In the past two decades, with the development of science and technology, further understandings have been made in the functional mechanisms and regulatory role in the innate immunity of SOCS proteins. At the same time, pathogens can also achieve innate immune escape by manipulating SOCS proteins and examples of viruses, bacteria and parasites have been found. Although many studies have been disclosed relationships between mRNA and protein levels of SOCS family and immune and inflammatory diseases, the regulatory mechanisms of expression levels of SOCS family members are still unknown. Due to the role of SOCS proteins in the immune response caused by viruses has not been fully elucidated, thus limiting the scientific and clinical advances made by immunologists and microbiologists. Therefore, further research on the function of SOCS proteins may reveal that these proteins have more unexpected effects in the immune system signaling pathway. These findings can also bring more scientific theoretical basis for the treatment of diseases.

## Author Contributions

SH carried out the literature search and exploration and completed most of the manuscript writing. RJ and AC helped with the completion of the manuscript and coordination of the project. KL, MW, MC, JH, DZ, SC, ML, XZ, YW, QY, SZ, XO, SM, QG, YY, BT, YL, LZ, ZY, BJ, and XC read and edited the manuscript. All authors contributed to the article and approved the submitted version.

## Conflict of Interest

The authors declare that the research was conducted in the absence of any commercial or financial relationships that could be construed as a potential conflict of interest.
